# Evidence for Conversion of Methanol to Formaldehyde in Nonhuman Primate Brain

**DOI:** 10.1155/2016/4598454

**Published:** 2016-03-15

**Authors:** Rongwei Zhai, Na Zheng, Joshua Rizak, Xintian Hu

**Affiliations:** ^1^Key Laboratory of Animal Models and Human Disease Mechanisms of the Chinese Academy of Sciences & Yunnan Province, Kunming Institute of Zoology, Kunming, Yunnan 650223, China; ^2^Kunming College of Life Science, University of the Chinese Academy of Sciences, Kunming 650204, China; ^3^University of the Chinese Academy of Sciences, Beijing 100049, China; ^4^CAS Center for Excellence in Brain Science and Intelligence Technology, Chinese Academy of Sciences, Shanghai 200031, China; ^5^Kunming Primate Research Center of the Chinese Academy of Sciences, Kunming Institute of Zoology, Kunming, Yunnan 650223, China; ^6^Yunnan Key Laboratory of Primate Biomedical Research, Kunming, Yunnan 650050, China

## Abstract

Many studies have reported that methanol toxicity to primates is mainly associated with its metabolites, formaldehyde (FA) and formic acid. While methanol metabolism and toxicology have been best studied in peripheral organs, little study has focused on the brain and no study has reported experimental evidence that demonstrates transformation of methanol into FA in the primate brain. In this study, three rhesus macaques were given a single intracerebroventricular injection of methanol to investigate whether a metabolic process of methanol to FA occurs in nonhuman primate brain. Levels of FA in cerebrospinal fluid (CSF) were then assessed at different time points. A significant increase of FA levels was found at the 18th hour following a methanol injection. Moreover, the FA level returned to a normal physiological level at the 30th hour after the injection. These findings provide direct evidence that methanol is oxidized to FA in nonhuman primate brain and that a portion of the FA generated is released out of the brain cells. This study suggests that FA is produced from methanol metabolic processes in the nonhuman primate brain and that FA may play a significant role in methanol neurotoxicology.

## 1. Introduction

Methanol, a single carbon alcohol, is an important public health and environmental concern because it leads to metabolic acidosis, visual impairment, central nervous system dysfunction, neurodegenerative conditions, and death [1–5]. Elevated methanol in the body can occur after accidental or intentional consumption and/or exposure to other exogenous methanol sources. Elevated methanol levels can also occur as a result of increased endogenous methanol production, such as in the generation of methanol through the hydrolysis of protein carboxymethyl esters, catalyzed either by methylesterases or through spontaneous nonenzymatic reactions [6].

Methanol metabolism and mechanisms responsible for its toxic actions in primates have been extensively investigated in the periphery. Typically, with respect to methanol metabolism in primates, there are three steps involved. The first step in the metabolic pathway is oxidation of methanol to formaldehyde (FA). An alcohol dehydrogenase (ADH) is primarily responsible for the initial step [7, 8]. The second step is the oxidation of FA to formic acid. A glutathione-dependent formaldehyde dehydrogenase specific for FA catalyzes the conversion of FA to formic acid [1]. Another formaldehyde dehydrogenase, which is NAD dependent, catalyzes this conversion in human erythrocytes [7, 8] and a high-activity aldehyde dehydrogenase is responsible for this conversion in liver mitochondria [9]. The third step is the oxidation of formic acid to carbon dioxide. 10-formyl-THF dehydrogenase, a ubiquitous enzyme in mammalian tissues, catalyzes this step [1, 10]. Notably, the rate of the final step is far lower in primates than it is in rodents [1, 11]. With respect to methanol toxicity, many studies have demonstrated that formic acid is primarily responsible for methanol's toxicity. For example, formic acid has been found to be responsible for the metabolic acidosis witnessed in methanol-intoxicated humans [12, 13] and nonhuman primates [14, 15] and the ocular toxicity observed in methanol-poisoned humans [12, 16] and nonhuman primates [17, 18].

Moreover, the toxic actions of methanol have also been reported in the brain of primates [2–4, 11, 19]. Given the fact that methanol is nonreactive [20] and less toxic than its metabolites [21], FA, the metabolic intermediate of methanol, was considered responsible for these effects because there is compelling evidence that suggests FA is related to AD pathology, both* in vivo* and* in vitro* [22–26].

While methanol metabolic processes in the brain of primates remains inexplicit, it is likely that the brain will use similar enzymatic pathways to metabolize methanol, as found in liver. While catalase has been reported to be expressed in human brain [27], the expression of ADH1 in primate brain has been controversial [7]. The expression of catalase provides a potential for the oxidation of methanol to FA in the primate brain, but no study has demonstrated this metabolic process through the direct evaluation of intracranial FA levels after injection of methanol into the brain of primates.

In this study, direct injections of methanol into the lateral ventricles of rhesus monkeys were carried out to directly investigate whether metabolic process of methanol to FA occurs in the brain of rhesus macaques. This approach allowed the direct investigation of methanol metabolic processes under precise control of dose to the animals' brain. The FA levels in CSF were then assessed at the different time points following a single methanol injection.

## 2. Materials and Methods

### 2.1. Animals and Treatment

All animal care and treatment in this study were performed in accordance with the guidelines for the national care and use of animals approved by the national animal research authority (China). All animal experiments were carried out after approval by the Institutional Animal Care and Use Committee (IACUC) of the Kunming Institute of Zoology.

Three 12-year-old male rhesus monkeys (*Macaca mulatta*) were recruited in this study. The body weights of the monkeys were as follows: Monkey #1 10.8 kg, Monkey #2 10.3 kg, and Monkey #3 11.4 kg. Each monkey was individually housed under standard laboratory conditions [28]. In order to provide the precise location of the right lateral ventricle and avoid interference caused by surgical operation in the results, as well as allowing animals to recover to stabilized FA levels, a surgical operation to implant a stainless steel tube into the right lateral ventricle was performed on each rhesus macaque prior to the experiment. Each animal was anesthetized with intramuscular atropine (20 mg/kg), ketamine (10 mg/kg), and sodium pentobarbital (20 mg/kg). The head of the animal was fixed in a stereotaxic instrument and the skull over the parietal lobe was exposed under aseptic conditions by a longitudinal skin incision followed by removal of the connective tissue. A small hole on the skull (<2 mm in diameter) was created with an electric drill at the following coordinates: anterioposterior (AP): interaural: 17 mm; mediolateral (ML): −2 mm. Then stainless steel tubing with a length of 40 mm (21-gauge, New England Small Tube Corporation, USA) was inserted into the right lateral ventricle (dorsoventral (DV) depth ranged from 18 to 22 mm). A successful puncture was judged by observing the cerebrospinal fluid (CSF) flowing out or CSF pulsations at the orifice. The outer portion of the stainless steel tube was then fixed on the skull with composite dental cement fixed to titanium nails screwed into the skull. After the operation, each monkey was intramuscularly injected with penicillin (1600 K Unit, Harbin Pharmaceutical Group Sixth Pharm Factory, Harbin, China) for at least seven days. All animals were allowed to recover after the surgery for more than two weeks.

Each monkey received a single injection of 200 *μ*L volumes of 5% (v/v) methanol in 0.9% (w/v) saline into lateral ventricle over a 15-minute period. After the injection, the needle was held in the place for 5 minutes. The methanol was purchased from Sigma (USA).

### 2.2. CSF Collection

In order to determine whether the level of FA in CSF was elevated following single intracerebroventricular (i.c.v.) methanol injections, the CSF from the methanol injected animals was collected at 0, 3, 6, 12, 18, 24, and 30 hours after the administration. The 0 hr refers to the point before methanol injection. Animals were anesthetized with ketamine (10 mg/kg) and approximately 0.5 mL of CSF was withdrawn through a lumbar puncture using a 22-gauge needle. Then the CSF samples were immediately frozen in liquid nitrogen and later transferred and stored in a −80°C freezer until analysis.

### 2.3. CSF Formaldehyde Measurements

Formaldehyde levels in the CSF following a single methanol injection were measured with the DFOR-100 formaldehyde detection kit as per the manufacturer's instructions (BioAssay Systems, Hayward, CA, USA). Briefly, CSF samples were deproteinated and neutralized prior to assaying. To deproteinate the CSF samples, 50 *μ*L of 10% TCA was added into each 100 *μ*L sample. Each sample was then vortexed and centrifuged at 14000 rpm for 5 min; 100 *μ*L of clear supernatant was transferred to a clean tube and mixed with 25 *μ*L of Neutralizer. Samples (50 *μ*L) were mixed with the DFOR reagent for 30 min and then assayed in a FlexStation 3 Multi-Mode Microplate Reader: *λ*_exc_ = 370 nm; *λ*_em_ = 470 nm.

### 2.4. Statistics

All statistical analyses were carried out with the GraphPad Prism 5 software. The levels of formaldehyde in the CSF were analyzed by analysis of variance with repeated measures followed by Tukey's test for intergroup difference. The level of significance was set at *p* < 0.05.

## 3. Results

**Figure 1:**
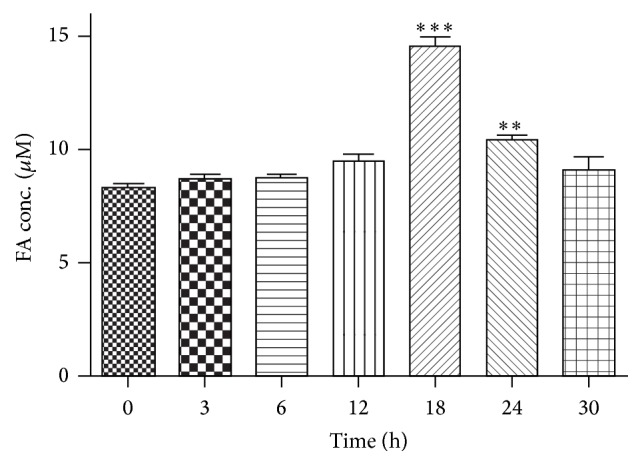
Formaldehyde (FA) levels in CSF samples following an i.c.v. injection of methanol in rhesus monkeys. FA levels in the CSF were measured at different time points. “0 h” refers to the point prior to the methanol injection. There was no significant difference until 18th hour after the methanol injection, albeit an increasing trend began after the 3rd hour. Data points represent the average CSF formaldehyde levels of the monkeys at each time point. All values are represented as the mean ± SEM. ^*∗*^ *p* < 0.05; ^*∗∗*^ *p* < 0.01; ^*∗∗∗*^ *p* < 0.001.

In order to investigate elevated levels of intracranial FA following methanol injections, CSF samples from monkeys given a single methanol injection were taken before administration (noted as “0” point) and at 3, 6, 12, 18, 24, and 30 hours after the injection. FA levels were then measured using a formaldehyde measurement kit. FA levels in the CSF following methanol treatments displayed an increasing trend and reached prominent differences compared to the “0” time point as a baseline at 18 and 24 hrs after the injection, respectively (Figure 1). The elevated FA levels returned to normal physiological levels at 30 hours (Figure 1). These findings indicated that endogenous methanol metabolism led to elevated intracranial FA levels in the brain of monkeys treated with methanol.

## 4. Discussion

Methanol is a natural chemical that poses dangers to human health. It can be found in cigarette smoke, canned fruits and vegetables, and aspartame-sweetened food products [20], as well as in beverages where drinking alcohol is inadvertently or criminally substituted with methanol [8]. Although methanol has been found to induce central nervous system dysfunction [4, 11] and neurodegenerative conditions [2, 3, 19], the mechanisms underlying its toxicity to the brain remain inexplicit in primates. The present study demonstrated that methanol could be oxidized to FA in primate brain and that a portion of the FA generated leaked out of the cells in which it was produced. This suggests that FA produced from methanol not only affects the cell in which methanol is metabolized but also may affect the surrounding tissue. It is noteworthy that formaldehyde levels in CSF present a gradual increasing trend which began at 3 hours following direct i.c.v. injection of methanol, although a significant elevation in FA levels only occurred after 18 hours. The time lapse of the first significant elevation in FA levels was dependent on (a) numbers of samples; (b) diffusion velocity of methanol into brain tissues; (c) metabolic capacity and speed of brain tissues to oxidize methanol to FA; (d) reactivity of produced FA to surrounding molecules; (e) diffusion velocity of FA from produced sites to CSF. Moreover, these results are consistent with formic acid data measured in primates, which suggests that methanol metabolism in the primate brain undergoes oxidation from methanol, via FA, to formic acid and carbon dioxide. FA is a significant consideration for human health because its toxicity is due to its high reactivity. FA readily attaches to proteins forming adducts or causes protein cross-linking by forming methylene bridges between amino groups [29, 30] and has the ability to damage DNA [31]. Elevated FA has been implicated in some neurodegenerative diseases. For example, elevated FA levels have been found in brains of patients suffering from neurodegenerative diseases like Alzheimer's disease (AD) or multiple sclerosis (MS) [22–24], where FA is known to cross-link proteins like tau (in AD) or myelin basic protein (MBP, in MS), which in turn results in the proteins losing their normal function and elicits an immune response that is characteristic of the diseases [20, 24]. It is noteworthy that some human subjects suffering from methanol poisoning develop symptoms of MS, which may be related to methanol oxidation to FA in brain that leads to MBP structure and function modification by the reactive FA [19].

Although FA is, without doubt, produced following methanol administration, it is not considered to be a toxic metabolite of methanol in the periphery. This is mainly due to FA being undetectable in the blood following methanol administration. This limited detection is likely due to its rapid metabolism to formic acid in the liver [32] and the blood [1] and because FA has a half-life of approximately 1.5 min in the blood of monkeys following its intravenous infusion [21]. This suggests that methanol metabolism through FA to formic acid in the periphery is rapid and that methanol toxicity might possess different mechanisms in the periphery and brain. The findings that methanol is converted to FA in the brain and found in the CSF after 18 hrs suggest that methanol toxicity may have deleterious effects in the CNS via FA.

## 5. Conclusion

In summary, elevated levels of intracranial FA were found in this study following a single methanol injection, which is the first demonstration of methanol oxidation to FA in the nonhuman primate brain. This study links the toxicity of methanol to its metabolites, FA and/or formic acid, in the brain.
